# COVID-19 SignSym – A fast adaptation of general clinical NLP tools to identify and normalize COVID-19 signs and symptoms to OMOP common data model

**Published:** 2020-07-13

**Authors:** Jingqi Wang, Huy Anh, Frank Manion, Masoud Rouhizadeh, Yaoyun Zhang

**Affiliations:** 1. Melax Technologies, Inc, 7000 Fannin St Suite 1900, Houston, TX, USA; 2. University of Michigan School of Nursing, 426 N Ingalls St, Ann Arbor, USA; 3. Johns Hopkins University School of Medicine, 2024 E Monument St. Baltimore, MD, USA

## Abstract

The COVID-19 pandemic swept across the world rapidly infecting millions of people. An efficient tool that can accurately recognize important clinical concepts of COVID-19 from free text in electronic health records (EHRs) will be significantly valuable to accelerate various applications of COVID-19 research. To this end, the existing clinical NLP tool CLAMP is quickly adapted to COVID-19 information. An automated tool called COVID-19 SignSym is built, which can extract signs/symptoms and their eight attributes (body location, severity, temporal expression, subject, condition, uncertainty, negation, and course) from clinical text. The extracted information is also mapped to standard clinical concepts in the common data model of OHDSI OMOP. Evaluations on clinical notes and medical dialogues demonstrate promising results. This tool is freely accessible to the community as a downloadable package of APIs (https://clamp.uth.edu/covid/nlp.php). We believe COVID-19 SignSym will provide fundamental supports to the secondary use of EHRs, thus accelerating the global research of COVID-19.

## Introduction

The COVID-19 pandemic^1^ swept across the world rapidly infecting more than two million people in the US and resulting in the death of almost 120,000 at a mortality rate of about 6%.^2^ Scientists and researchers from multiple organizations world widely have been working collaboratively targeting effective prevention and treatment strategies.^3^ Research findings, data resources, informatics tools and technologies are being openly shared, aiming to speed up the fight against such emerging pandemic.^4,5^

Facilitated by PubMed, large datasets of literature articles relevant to COVID-19 are being accumulated and shared at a rapid pace in the medical community.^6^ For example, the COVID-19 Open Research Dataset (CORD-19) has already accumulated more than 75, 000 full text articles.^6^ Based on such resources, many tools have been developed using natural language processing (NLP) techniques to unlock COVID-19 information from literature, including tools of search engines, information extraction and knowledge graph building.^7,8^ As another important data source for COVID-19 research, Electronic Health Records (EHRs) store the clinical data of COVID-19 patients, which are critical for various applications, such as clinical decision support, predictive modeling and phenotyping-based cohort stratification.^9,10^ An efficient tool that can accurately recognize important clinical concepts of COVID-19 from clinical notes will be significantly valuable to save time and accelerate the chart-review workflow.

Despite that several large consortia have been formed to construct large clinical data networks for COVID-19 research, such as The National COVID Cohort Collaborative (N3C)^11^ and the international EHR-derived COVID-19 Clinical Course Profiles (4CE)^12^, very few informatics tools have been developed for clinical notes of COVID-19. As far as the authors know, the only tool available in public is MedTagger.^13^ Based on a pre-collected list of symptoms and synonyms, MedTagger recently provides a rule-based tool, which mainly extracts COVID-19 sign/symptoms and their three attributes of certainty, status (i.e., HistoryOf or Present) and experiencer (i.e., Patient or others).^14^ However, limited sign/symptom lists may not be sufficient to recognize or normalize the varying expressions in clinical text; other important attributes critical to unfold the clinical course and prognosis of patients are also not recognized in this tool, including the onset time, severity, course and body location. On the other hand, some existing clinical NLP tools can already recognize signs/symptoms and a more comprehensive set of attributes with good performances. Given that COVID-19 signs/symptoms are a subset of a more general scope of clinical problems, existing tools can be leveraged and quickly adapted for COVID-19 information extraction.

Therefore, in this study, we built an automatic NLP tool, named as COVID-19 SignSym, to extract COVID-19 signs/symptoms and their eight attributes (body location, severity, temporal expression, subject, condition, uncertainty, negation, and course), by adapting existing pipelines in the CLAMP software (Clinical Language Annotation, Modeling, and Processing Toolkit).^15,16^ The extracted entities will also be normalized to standard terms in OHDSI OMOP CDM (Observational Medical Outcomes Partnership, Common Data model)^17^ automatically. The set of signs and symptoms of COVID-19 is collected from four resources, including the case record form of WHO (World Health Organization), National COVID Cohort Collaborative, dictionaries in MedTagger and in-house dictionaries from John Hopkins. In total, 55 signs and symptoms are collected from these sources. UMLS CUIs of these signs and symptoms are assigned manually, their synonyms in UMLS are also extracted to extend the dictionary. A hybrid method combining deep learning models, lexicons and pattern-based rules is used to build COVID-19 SignSym. We believe this tool will provide fundamental supports to the secondary use of EHRs, thus accelerating the global research of COVID-19.

## Methods

**Figure 1.**
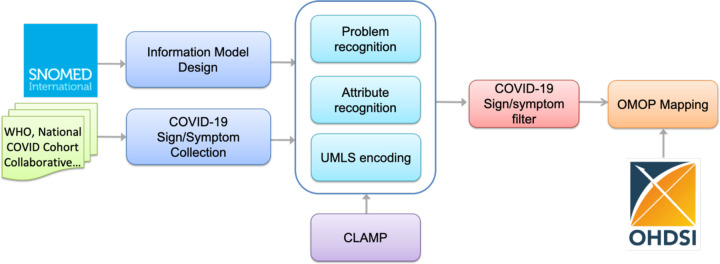
An overview of the NLP pipeline for COVID-19 sign/symptom extraction and normalization

Figure 1 illustrates an overview of the workflow for building COVID-19 SignSym. This workflow mainly consists of five steps: (1) Information model design to define the information scope of COVID-19, i.e. semantic types of clinical concepts and relations, to be extracted; (2) Sign/symptom collection to gather COVID-19 signs/symptoms from multiple sources; (3) Information extraction and normalization to extract and normalize COVID-19 signs/symptoms and eight types of attributes, including body location, severity, temporal expression, subject, condition, uncertainty, negation, and course. Hybrid approaches are used to adapt existing NLP pipelines in CLAMP for COVID-19 information extraction. Lexicons of COVID-19 signs/symptoms and pattern-based rules are integrated with CLAMP to optimize the performance. All signs/symptoms in free text are extracted first, which are filtered by lexicons and UMLS CUIs to maintain COVID-19 signs/symptoms. (4) OMOP Mapping to convert the output information into the format of OMOP CMD.

### Dataset

Four clinical datasets were used for building and evaluating the pipeline:

1. MIMICIII: this data set contains clinical notes from intensive patient care. 200 discharge summaries are randomly selected, with 50% for model tuning and 50% for open test. In total, the selected dataset contains 17, 208 sentences and 329, 044 tokens.
2. Medical dialogues: this data set contains medical dialogues related to COVID-19 collected from an online website between patients and doctors.^18^ Fifty dialogues were randomly selected for external evaluation of COVID-19 SignSym. In total, the selected dataset contains 1,162 sentences and 22,324 tokens.
3. Clinical notes from Johns Hopkins: This dataset contains 334 clinical notes of 40 patients and includes relevant note types such as H&P, Critical Care Notes, Progress Notes, and ED Notes, focusing specifically on the notes created within 48 hours before and after hospital admission. Notes are pre-processed by Hopkins in-house section identification tools, and only the relevant narrative parts, particularly the chief complaint and history of the present illness sections are extracted. For each of the 40 patients, each symptom was labeled as present or not-present, resulting in over 467 manually annotated symptoms. These gold standard labels are then used to validate COVID-19 SignSym. In total, the clinical notes in this dataset contain 13,397 sentences and 121,802 tokens.

### Information model

**Figure 2.**
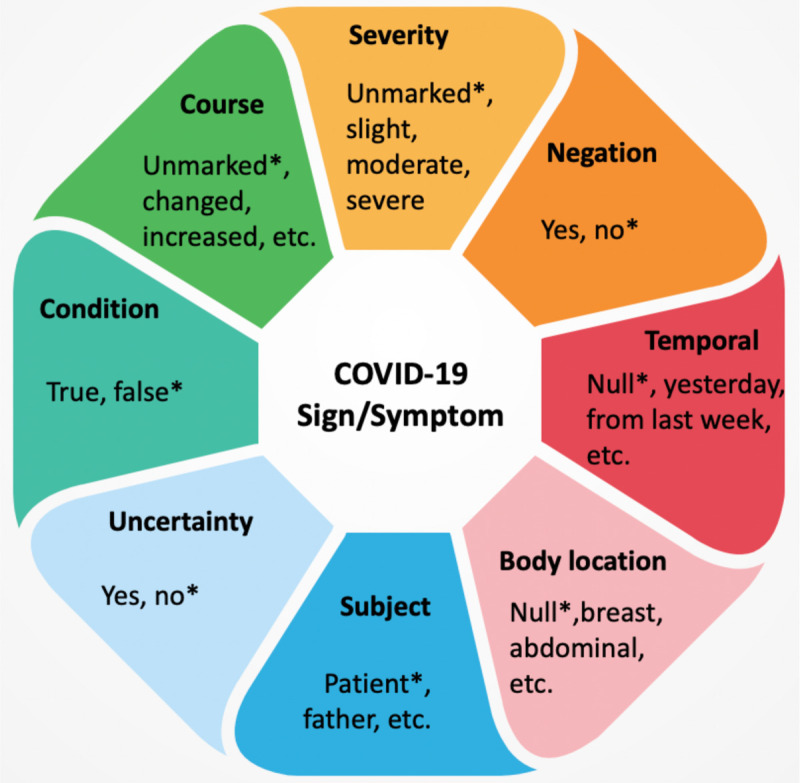
Information model of COVID-19 signs/symptoms and their attributes.

The information model followed by the COVID-19 SignSym is illustrated in Figure 2. In addition to mentions of signs and symptoms, eight important attributes and their relations are also recognized: (1) Severity: indicates the severity degree of a sign/symptom; (2) Negation: indicates a sign/symptom was negated; (3) Temporal information: indicates the time period or specific time the sign/symptom started; (4) Subject: indicates who experienced the sign/symptom; (5) Uncertainty: indicates a measure of doubt into a statement about a sign/symptom; (6) Condition: indicates conditional existence of sign/symptoms under certain circumstances; (7) Body location: represent an anatomical location of the sign/symptom; (8) Course: indicates progress or decline of a sign/symptom. Examples of attribute values are illustrated in Figure 2. * indicates their default values.

### Lexicon building for COVID-19 signs and symptoms

Leveraging the community efforts, COVID-19 signs and symptoms are collected from five sources: (1) WHO case record form: 26 signs and symptoms were collected from the SIGNs AND SYMPTOMS ON ADMISSION section of the case record form provided by WHO.^19^ (2) National COVID Cohort Collaborative: 15 signs and symptoms were collected from the diagnosis table shared by the national COVID cohort collaborative as phenotyping information.^11^ (3) MedTagger Lexicon: 17 signs and symptoms together with their 136 synonyms were collected from the lexicon in MedTagger.^14^ (4) Lexicon from Johns Hopkins: 14 signs and symptoms, and 337 synonyms were collected from an in-house lexicon of Johns Hopkins. UMLS CUIs of these signs and symptoms (in total 124) were also assigned manually, with their UMLS synonyms collected. After removing redundancy, 55 signs and symptoms and 2, 022 synonyms were collected from these four sources. Table 1 illustrates ten signs/symptoms of COVID-19, together with their CUIs and example synonyms. A comprehensive list is included in Supplementary Table 1.

### Text processing

#### Disease-attributes pipeline in CLAMP:

The disease-attribute pipeline in CLAMP was adapted for COVID-19 information extraction in this study. This pipeline is built to automatically extract mentions of problems and their eight attributes from clinical text. The definition of problems follows that used in the i2b2 2010 shared task, which consists of eleven semantic types in UMLS (e.g., sign or symptom, pathologic functions, disease or syndrome, etc.).^20^ Besides, the definitions of attributes follows that used in the SemEval 2015 Shared Task 14.^21^

#### Adapting CLAMP for COVID-19 Sign/Sym:

Besides, the lexicons collected previously are also used in an additional step of dictionary-lookup, to improve the coverage of recognized COVID-19 signs and symptoms. Furthermore, regular expressions and rules are also applied in a postprocessing step to boost the performance of attribute recognition.

#### Concept normalization

Mentions of problems and attributes are normalized to standard concepts using the UMLS encoder module of CLAMP, which are also built on a hybrid method of semantic similarity based ranking and rules of concept-prevalence in UMLS.^22^ Both CUIs and preferred terms in UMLS will be output for each recognized entity.

#### Filtering of COVID-19 Signs/Symptoms:

Once medical problems are automatically recognized and normalized to UMLS CUIs, they will be filtered by the pre-collected lexicons and CUIs of COVID-19 signs and symptoms.

#### OMOP Mapping

The remaining signs/symptoms and their attributes will also be mapped to standard concepts in OMOP CDM. The OMOP encoder module of CLAMP is used for this purpose, which applies a similar approach as in the UMLS encoder module, with a different scope of standard concepts and identifiers.

### Evaluation

#### Evaluation criteria:

(1) The performances of NER and relation extraction are evaluated using precision, recall and F-measure (F1); (2) The performance of concept normalization is evaluated using accuracy; (3) The performance of patient-level COVID-10 diagnosis is evaluated using precision, recall and F-measure.

#### Evaluation setup:

(1) 200 discharge summaries are randomly selected from MIMICIII; 100 of them are used for error analysis and optimize the SignSym pipeline. After that, the information extraction performance of COVID-19 SignSym are evaluated on another 100 discharge summaries from MIMICIII as the open test. In addition, it is evaluated on fifty posts of COVID-19 related dialogues between patients and doctors online as an external test. (2) Besides, 1, 000 output CUIs are randomly selected from the 100 discharge summaries and manually reviewed to evaluate the performance of clinical concept normalization. (3) Moreover, a use case of identifying patients symptoms at presentation from notes generated within 48 hours before and after hospital admission is used to validate the effectiveness of COVID-19 SignSym. Specifically, positive and negative signs and symptoms in 334 clinical notes of 40 patients from Johns Hopkins Hospitals are extracted, and then aggregated into patient-level scores to determine all presenting symptoms for each patient. In total, 467 unique signs and symptoms are manually annotated and normalized for the 40 patients.

## Results

### Information extraction:

Performances of COVID-19 SignSym for information extraction are illustrated in Table 2; both performances on clinical text and medical dialogues are reported. The 95% confidence intervals (CIs) are also reported in Table 2, by considering each clinical note or each post of medical dialogues as a sample. Promising results were achieved on sign/symptom extraction, with a F-measure of 0.992 ± 0.008 on clinical text and 0.99 ± 0.01 on medical dialogue. As for recognizing attributes of signs/symptoms, the tool yielded better performances on clinical text than on medical dialogue (e.g., F-measure: Has_Body location 0.986 ± 0.014 vs. 0.964 ± 0.036, Has_Temporal 0.984 ± 0.016 vs. 0.926 ± 0.074). Some semantic types have low frequencies in the test datasets, thus have large CIs. For example, there are only six instances of Subject (F-measure: 0.771 ± 0.229) in clinical notes and 3 instances of Course in the medical dialogues (F-measure: 0.579 ± 0.421), leading to large CIs.

### Concept Normalization:

Based on manual check of 1, 000 gold standard entities and their automatically assigned CUIs, COVID SignSym obtained an accuracy of 95% for concept normalization.

### COVID-19 sign/symptom presentation of patients:

In comparison with manually assigned signs/symptoms of each patients, the tool yielded a precision of 0.928, a recall of 0.957, and a F-measure of 0.942.

### Tool Availability

**Figure 3.**
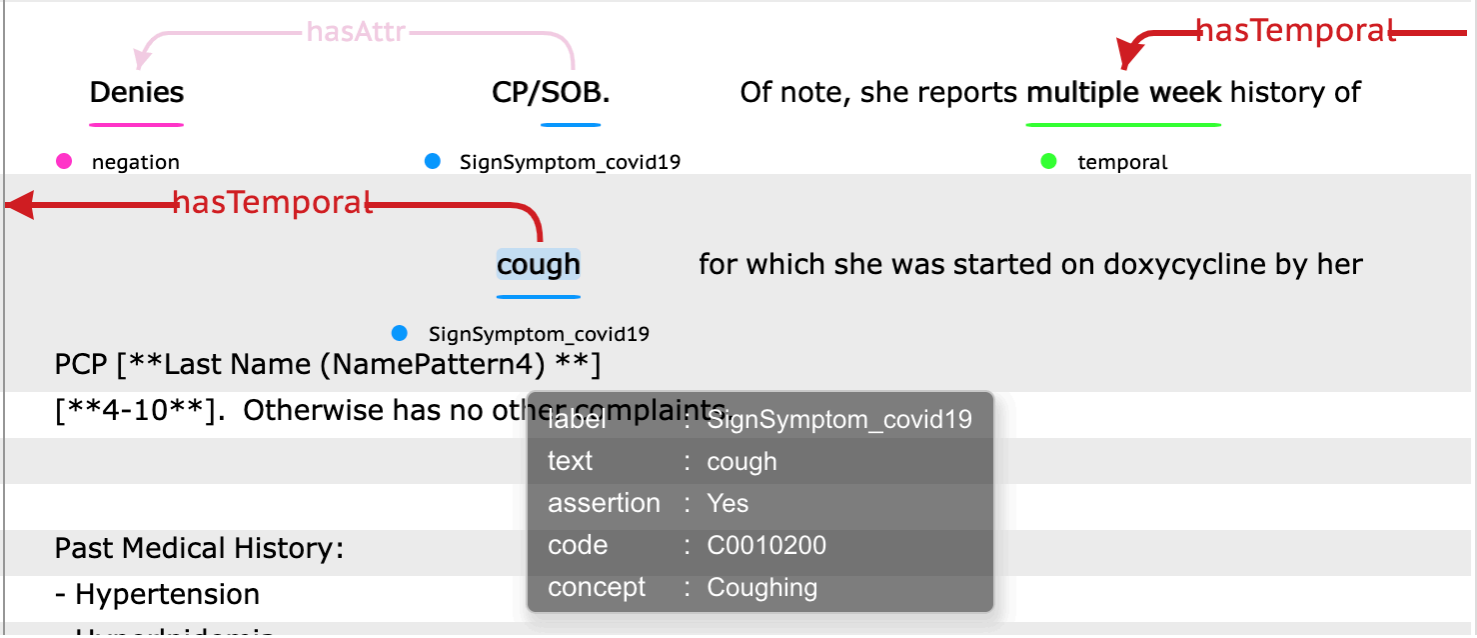
An output illustration of the COVID-19 sign/symptom extraction tool

It is freely accessible to the community via a downloadable package of APIs^19^. A visualization of the output format is illustrated in Figure 3.

## Discussion

There is an urgent demand of automatic tools that can extract COVID-19 information from clinical text. This paper presents our preliminary work to quickly adapt an existing high-performance clinical NLP tool for COVID-19 sign/symptom extraction and normalization, leveraging COVID-19 lexicons provided from multiple resources. The tool of COVID-19 SignSym is evaluated on clinical notes and medical dialogues. Experimental results demonstrate that it achieves promising results in practical settings. The workflow of this study can be generalized to other use cases, where existing clinical NLP tools need to be customized for specific information needs within a short time.

Errors present in the outputs of COVID-19 SignSym are analyzed carefully for further improvement. One type of common errors is partial recognition of named entities. For example, “cough” is recognized, instead of “cough productive of yellow sputum”. More patterns of signs and symptoms are needed to improve the coverage. Another type of common errors is related to cross sentence relations. For example, many temporal expressions are not in the same sentence with the relevant signs/symptoms. Besides, some attributes such as conditions are modifying signs/symptoms in a list of multiple items. Document structure and intra sentence relations need to be handled in the next step.

Limitations and future work: this work has several limitations and future works are needed. (1) First, performances on two COVID-19 datasets are evaluated and reported, additional evaluations are needed to further refine the tool and increase its generalizability; (2) Currently, only sign/symptoms and their attributes are extracted, additional works will be conducted for more related information such as comorbidities and medications; (3) The output information will also be mapped to other clinical data standards such as FHIR in the near future, to facilitate clinical operations and other applications.

## Conclusion

This paper presents an automatic tool, named as COVID-19 SignSym, to extract and signs/symptoms and their eight attributes from clinical text using NLP techniques. SignSym achieved promising results on clinical notes and medical dialogues. It is freely accessible to the community via a downloadable package of APIs. So far, COVID-19 SignSym has applied by eight different institutions in academia and industry, which we believe will fundamental supports to the secondary use of EHRs for COVID-19 research upon a dissemination.

## Figures and Tables

**Table 1:** Ten Examples of COVID-19 Signs and Symptoms, their synonyms and UMLS CUIs

Sign and Symptom	Example Synonym	UMLS CUI
Sore throat	throat pain, throat soreness	C0242429
Headache	head pain, cephalalgia	C0018681
Fever	Hyperthermia, febrile	C0015967
Fatigue	Tired, energy loss	C0015672
Abdominal pain	stomach pain, gut pain	C0000737
Altered consciousness	Consciousness Disturbances, Impaired consciousness	C0234428
Short of breath	Sob, gasp, Dyspnea	C0013404
Dry cough	cough unproductive, nonproductive cough	C0850149
Vomiting	throw up, puke	C0042963
Diarrhea	loose stool, watery stool	C0011991

**Table 2.** Information extraction performances of COVID-19 SignSym on clinical text and medical dialogues. 95% confidence interval is reported for each result.

	Clinical text			Medical dialogue		
	Precision	Recall	F1	Precision	Recall	F1
Sign/Symptom	0.98 ± 0.01	0.985 ± 0.009	0.992 ± 0.008	0.967 ± 0.015	0.988 ± 0.009	0.99 ± 0.01
Attribute extraction						
Has_Body location	0.982 ± 0.017	0.95 ± 0.03	0.986 ± 0.014	0.933 ± 0.058	0.883 ± 0.076	0.964 ± 0.036
Has_Temporal	0.916 ± 0.067	0.97 ± 0.03	0.984 ± 0.016	0.83 ± 0.081	0.849 ± 0.078	0.926 ± 0.074
Has_Negation	0.976 ± 0.024	0.961 ± 0.034	0.991 ± 0.009	0.923 ± 0.077	0.831 ± 0.144	0.968 ± 0.032
Has_Condition	0.949 ± 0.051	0.949 ± 0.051	0.99 ± 0.01	0.874 ± 0.117	0.799 ± 0.145	0.939 ± 0.061
Has_Course	0.926 ± 0.074	0.894 ± 0.105	0.989 ± 0.011	0.646 ± 0.354	0.646 ± 0.354	0.579 ± 0.421
Has_Uncertainty	0.941 ± 0.059	0.889 ± 0.103	0.983 ± 0.017	0.82 ± 0.166	0.785 ± 0.181	0.932 ± 0.068
Has_Severity	0.926 ± 0.074	0.926 ± 0.074	0.926 ± 0.074	0.84 ± 0.158	0.801 ± 0.183	0.952 ± 0.048
Has_Subject	0.771 ± 0.229	0.771 ± 0.229	0.771 ± 0.229	0.860 ± 0.13	0.753 ± 0.164	0.923 ± 0.077
